# Selections

**Published:** 1897-04

**Authors:** 


### Selections.

### PEROXIDE OF HYDROGEN IN WOUND TREATMENT.

Dr. Neudorffer recommends a two-and-a-half-per-cent. solution of peroxide of hydrogen as a hæmostatic and disinfectant in the treatment of wounds. Through the action of the hydrogen peroxide the fibrin of the blood is separated in minute microscopic fibres, resulting in a local defebrinization of the blood, whereby the harmless little clots close the wounded surface from the surrounding tissue.

The peroxide of hydrogen solution is applied by means of pledgets of absorbent cotton, which are well pressed, leaving them only damp; in this manner they are applied to the bleeding surface only but a second.

Peroxide of hydrogen, according to Neudorffer, may also be used in severe cases of hemorrhage, as bleeding from the nose, where a simple sponging or wiping of the nasal cavities or passages with a two-and-a-half-per-cent. solution usually checks the bleeding; or a similar result may be obtained by inhaling the solution five or six times through the nostrils in the form of a spray, taking deep inhalations at the time.

